# Dynamic distribution and prevention of spontaneous combustion of coal in gob-side entry retaining goaf

**DOI:** 10.1371/journal.pone.0267631

**Published:** 2022-05-27

**Authors:** Dongjie Hu, Zongxiang Li

**Affiliations:** 1 College of Safety Science and Engineering, Liaoning Technical University, Fuxin, Liaoning, China; 2 Key Laboratory of Mine Thermodynamic disasters and Control of Ministry of Education, Liaoning Technical University, Fuxin, Liaoning, China; Tianjin University, CHINA

## Abstract

The 11101 working face of Qipanjing Mine was taken as the research object to explore the dynamic change law of the spontaneous combustion of the remaining coal in the gob-side entry retaining goaf area. A sealed oxygen consumption experiment was conducted to determine the (critical) oxygen volume fraction in the suffocation zone and continuous oxygen consumption rate of coal samples. The parameters of the working face were measured on site, and the air volume fraction in the goaf was monitored using a beam tube. Considering upward ventilation and the effect of gravity, a UDF control program for the falling medium in the gob-side entry retaining goaf was written. Based on the experimental results, a control program for the continuous oxygen consumption rate of the remnant coal was compiled, the dynamic distribution of the flow field in the gob-side entry retaining goaf was simulated with different advancing positions and air leakage at the working face, and a prediction model for the spontaneous combustion danger area was established. Finally, fire prevention measures via grouting in the return air lane side and nitrogen injection in the retaining lane side were put forward. The results showed that with the variation in the advancing position of the working face or in the air leakage of the air intake lane, the range of the natural hazardous area of the gob-side entry retaining goaf presents a distribution with a power function *S*_*F*_ = *x*^*n*^*+b* (0 < *n* < 1). The theoretically proposed fire-fighting measures can effectively reduce the risk of spontaneous combustion of coal.

## I. Introduction

China’s economy is transforming toward high-quality development, placing higher demands on coal companies in terms of efficient supply, energy conservation, and emission reduction [1, 2]. In recent years, many scholars have verified the feasibility of the gob-side entry retaining mining technology in implementing supply-side reforms and ensuring efficient and sustainable development of mining enterprises [3–5]. However, the application conditions of this technology are harsh, and field application is often restricted by the spontaneous combustion of the leftover coal, which has become one of the main reasons hindering its promotion.

In 2017, Manchao proposed a new green and efficient coal mining method, called the “110” method, ushering in the third revolution in mining science and technology [6]. Scholars have studied the spontaneous combustion characteristics of coal in the goaf from much earlier, and many relevant laws have been revealed to determine a method for judging the risk of spontaneous combustion in the goaf [7–10]. Wenhu pointed out five parameters for the spontaneous combustion of floating coal in the goaf. The three zones of the goaf spontaneous combustion are in a dynamic and stable state, and the risk of spontaneous combustion in the goaf can be predicted in advance from the temperature distribution [11]. Minggao used MATLAB software to superimpose the measured O_2_, CO_2_, and CH_4_ concentrations and temperature to determine the area of the spontaneous combustion danger zone in the goaf [12]. The above research revealed many relevant laws and determined a method to judge the risk of spontaneous combustion of coal in the goaf; in practical applications, an oxygen volume fraction of 10% or 8% is typically used as the basis for classifying the spontaneous combustion zone. However, the spontaneous combustion characteristics of different types of coals are different, and the corresponding values are also different [13]. Although the spontaneous combustion mechanism of the leftover coal in the goaf is the same, because there is no coal pillar for mining along the goaf, the goaf in the upper section and the goaf behind the current working face will be connected during mining. The air leakage at the working face and in the air intake lane on the side of the roadway affects the spontaneous combustion of the remaining coal in the entire goaf [14–16]. Unlike the U-shaped ventilation in coal pillar mining, the gas flow field distribution in the gob-side entry retaining goaf changes dynamically with the advancement of the working face. The goaf area behind the working face is close to the spontaneous combustion oxidation zone on the side of the return airway. It is impossible to judge the degree of risk of spontaneous combustion by continuously extending to the depth. The spontaneous combustion hazard characteristics of the gob-side entry retaining goaf are relatively complicated, with currently few studies in this area.

Hence, to more accurately judge the risk of spontaneous combustion of coal in the gob-side entry retaining goaf of the 11101 working face of Qipanjing Mine, a closed oxygen consumption experiment was conducted to study the oxygen consumption characteristics of coal samples taken from the mine and to determine their continuous oxygen consumption rate and critical oxygen volume fraction in the suffocating zone [17]. The objective was to study the dynamic relationship between the spontaneous combustion hazard characteristics of the gob-side entry retaining goaf and the different advancing positions of the working face and to propose targeted fire-fighting measures. To meet this objective, based on on-site measured data, the boundary conditions and gas emission sources of a goaf simulation model for seven groups of working faces at different advancing positions were set up. Combined with the closed oxygen consumption experiment, a control program for the oxygen consumption rate of the residual coal with the continuous change in the oxygen volume fraction in the simulation calculation was written. Based on the field observation and analysis, it is concluded that the caving of the surrounding rock in the goaf is more suitable for “∩+∪” type. A control program for the surrounding rock caving of the goaf in the simulation model was written based on the “O”-type ring theory. Through a simulation, the dynamic distribution of the gas flow field in the gob-side entry retaining goaf with the advancement of the working face was obtained, and combined with the sealed oxygen consumption experiment, the spontaneous combustion danger area was determined. Based on the simulation results, a grouting method in the return airway that can help judge the risk of spontaneous combustion in the gob-side entry retaining goaf and a method for injecting nitrogen on the side of the roadway that can help reduce the risk of spontaneous combustion in the entire goaf were proposed.

## II. Sealed oxygen consumption experiment of 9–1 coal sample

With the expansion of the goaf area, the oxygen supplement and temperature reduction of air leakage in the goaf gradually decreases to zero; therefore, it can be concluded that the oxidation of the deep goaf residual coal proceeds in a closed state. A closed oxygen consumption experimental device can help simulate the environment of a deep abandoned coal in mined-out areas. In this study, the main parameters determined using such an experimental device are (1) the critical oxygen volume fraction of coal sample 9–1 in Qipanjing Mine, and (2) the oxygen consumption rate of the coal sample with the continuous decrease in the oxygen volume fraction.

**Fig 1:**
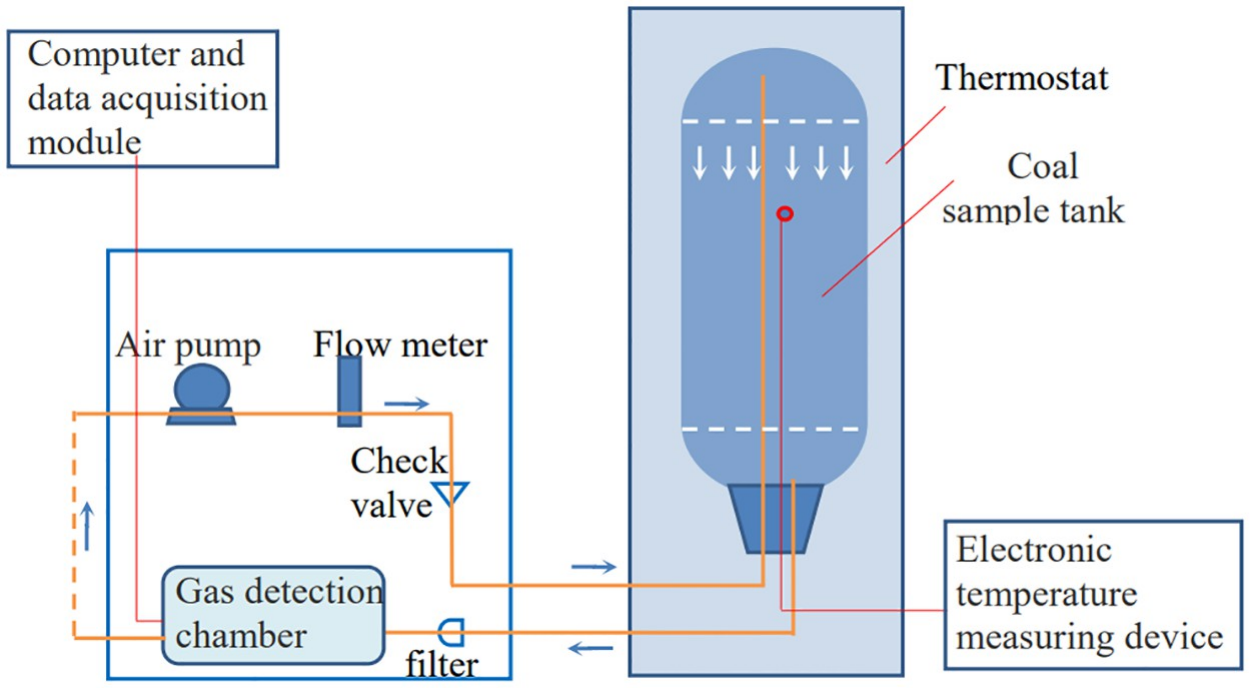
Schematic of sealed oxygen consumption experimental device.

The experimental device mainly comprises a computer, an air pump, a gas concentration sensor, a special data collector, and other interrelated parts. Fig 1 shows the internal pipeline connection of the device, where the coal sample tank is at standard atmospheric pressure. The preparation process for the experiment is as follows. The air inlet pipe and temperature detector are inserted into the coal sample tank after cleaning and drying, and the coal sample to be tested is placed in the tank after crushing and screening for the specific size of coal particles. The tank is sealed to ensure that it does not leak. Before the experiment is started, air is sent for a short period of time to wash the coal sample gas to eliminate the interference of other gases, running incubator for its stable at 20°C. The data acquisition software records the O_2_ and CO concentrations in the process of the experiment by regulating the coal temperature, the temperature control system after being leveled off data curve of coal sample can be critical of smothering extinguish the oxygen density. At this point, the experiment can be terminated.

Therefore, a sealed oxygen consumption experiment was used to measure the critical oxygen volume fraction of the coal samples in the 11101 working face of Qipanjing Mine.

### 1. Critical oxygen volume fraction of the suffocating zone

Air leakage is a necessary condition for the spontaneous combustion of coal that remains in the goaf. The oxidation reaction between the leftover coal and the oxygen in the leaked air will develop into a spontaneous combustion under suitable heat storage conditions. According to the theory of coal oxidative spontaneous combustion, the oxygen concentration in the air leakage airflow is the decisive factor for the spontaneous combustion. Therefore, it is reliable to use the oxygen volume fraction to divide the “three zones” of the oxidative spontaneous combustion in the goaf. When dividing the “three zones” by the oxygen volume fraction, most scholars adopt 10% (or 8%) as the critical oxygen volume fraction to determine the oxygen extinguishing zone. However, due to the different spontaneous combustion characteristics of different types of coals, the corresponding values are different. Therefore, if 10% (or 8%) is uniformly used to judge the spontaneous combustion risk in the goaf, it will be evidently inaccurate and will have little guiding role in the safe and efficient production of coal.

**Fig 2:**
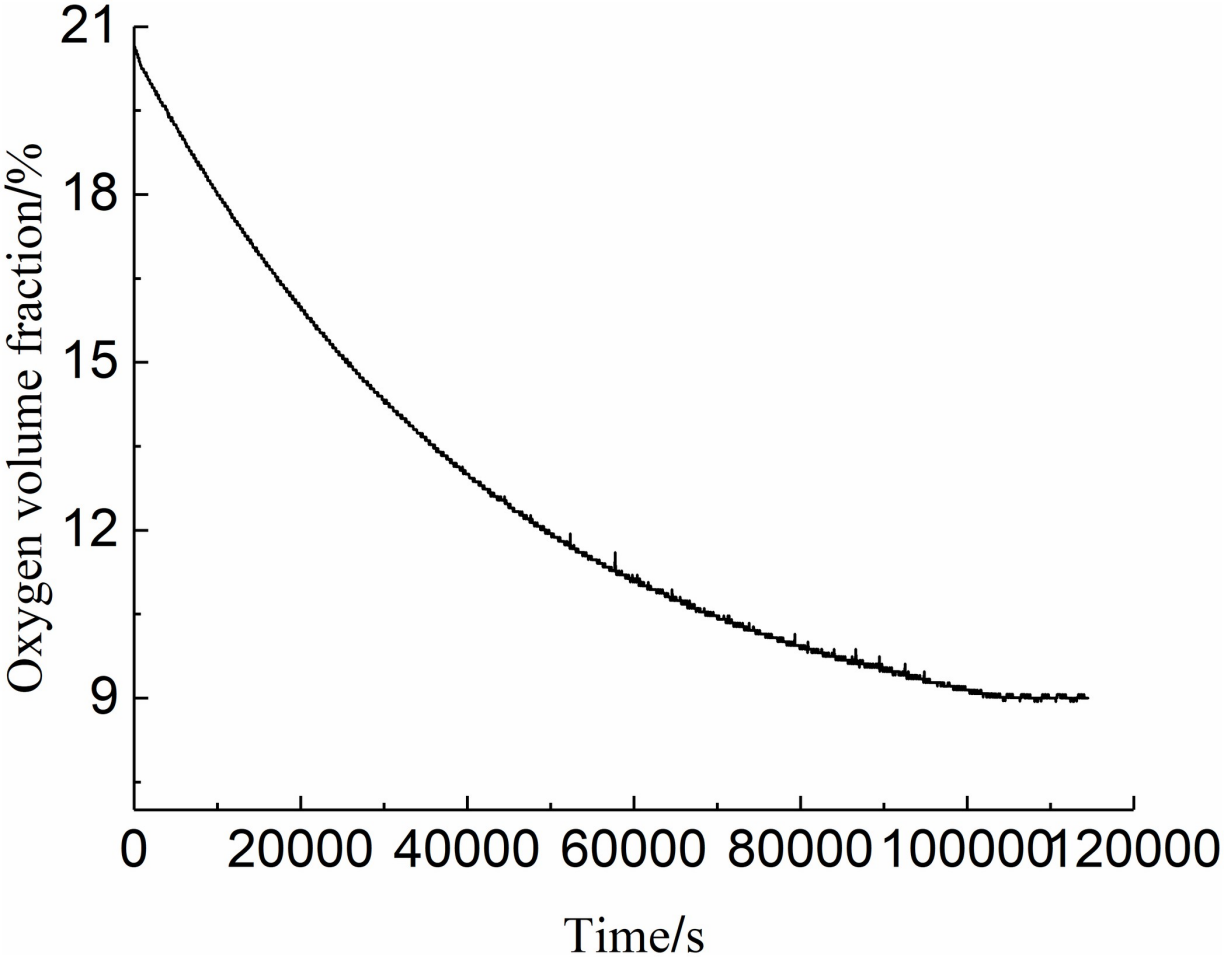
Experimental curve of closed oxygen consumption of Qipanjing coal sample.

After the experiment, the oxygen consumption curve of the checkerboard well coal sample in the closed oxygen consumption experiment is shown in Fig 2. As shown, the oxygen volume fraction in the closed device tends to stabilize as the experiment progresses, and the corresponding ordinate value of 9% is the (critical) oxygen volume fraction value of the checkerboard well suffocating zone^13^. When the oxygen volume fraction is greater than 9%, the residual coal is likely to spontaneously ignite. Therefore, the area with an oxygen volume fraction greater than 9% in the goaf is called the spontaneous combustion risk area.

### 2. Continuous oxygen consumption rate of 9–1 coal sample

The oxygen consumption characteristics of the coal samples under different oxygen concentrations are different [18–20]. Therefore, with the gradual decrease in the oxygen concentration in a deep goaf, the rate of oxygen consumption of the residual coal also constantly varies. The experimental device reported herein can not only measure the oxygen volume fraction of the coal sample to determine the boundary position of the oxygen extinguishing zone, but can also obtain the oxygen consumption rate under the continuous change in the oxygen concentration. This value plays a significant role in simulating the flow field distribution in the goaf using the fluid simulation software.

The principle of the algorithm for extracting the experimental parameters is as follows: Assuming that the oxygen concentration volume fraction C(τ) in the sealed tank approximately obeys the distribution of the negative exponential function:

$$c\left(\tau \right)=c_{\text{b}}+\left(c_{\text{0}}-c_{\text{b}}\right)\cdot e^{-\lambda c\tau }.$$ (1)

*C*_0_: Initial volume fraction of oxygen, %;

*λ*_*c*_: Decay rate of the oxygen volume fraction, s^−1^;

*C*_b_: Stable oxygen volume fraction value, %;

*τ*: oxidation time, s.

By taking the derivative of Eq (1) with time *τ*, the mass change rate of the oxygen mole consumed by the coal samples per unit time and volume in the closed tank can be obtained:

$$\gamma =\{\begin{array}{c} 0\\ -\text{0.4464}\lambda c\text{(}c_{\text{0}}-c_{\text{b}}\text{)}e^{-\lambda_{c}\tau } \end{array}\begin{array}{c} ,\\ , \end{array}\begin{array}{c} \left(c(\tau )<c_{b}\right)\\ \left(c(\tau )\geq c_{b}\right) \end{array}.$$ (2)

44.46: Amount of substance per unit volume, mol·m^−3^;

*γ*: Volumetric oxygen consumption rate, mol·(m^3^·s)^−1^.

Substituting Eq (1) into Eq (2) yields the following:

$$\gamma =-0.4464\lambda_{\text{c}}\left[c\left(\tau \right)-c_{b}\right].$$ (3)

Eq (3) shows that the oxygen consumption rate of the coal sample tank is directly proportional to the oxygen volume fraction (linear relationship). It can be inferred that the oxygen consumption rate is directly proportional to the oxygen volume fraction for the coal in the loose accumulation state.

Thus, the volumetric oxygen consumption velocity constant of the coal sample tank is obtained when the oxygen volume fraction is *C*_0_:

$$\gamma_{\text{0}}=-0.4464\lambda_{\text{c}}\left(c_{0}-c_{b}\right).$$ (4)

*γ*_0_: Volumetric oxygen consumption velocity constant of the initial coal sample.

## III. Working face profile and site data

**Fig 3:**
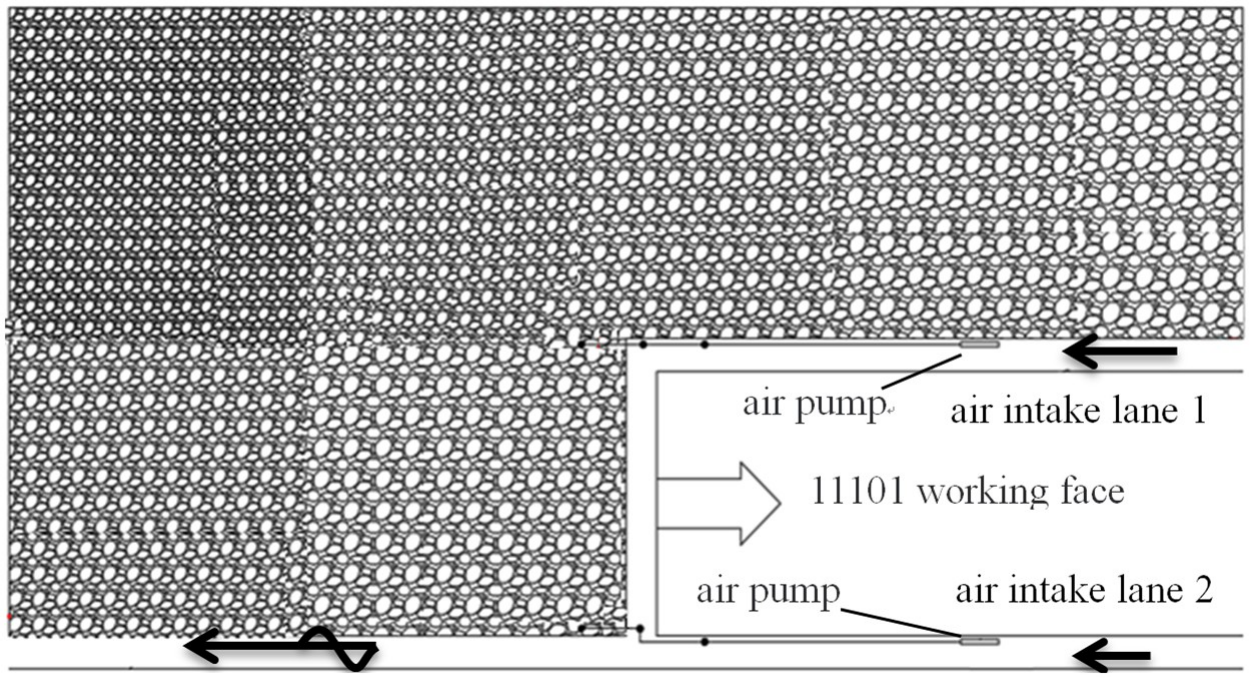
Schematic of the “three zone” distribution of the goaf in Qipanjing Mine.

The average coal thickness in the mining range of the 11101 working face is approximately 3 m, the incline length of the working face is 200 m, the height is 3 m, and the width is 5 m. The advancing length of the working face is approximately 2000 m. The results of < Prediction of Mine gas Emission and Gas Grade Evaluation Report > and < Identification Report of Coal Spontaneous Combustion Tendency > by Chongqing Research Institute showed that the maximum relative gas emission is 4.01 m^3^/t and that the maximum absolute gas emission is 10.31 m^3^·min, indicative of a low gas mine. The grade of the spontaneous combustion tendency of coal seam 9–1 is II, which makes it a spontaneous combustion coal seam. The shortest spontaneous combustion period of the coal seam where 11101 working face is located is 66 days. The gob-side entry retaining goaf in Qipanjing Mine includes the upper and rear goafs of the current working face. Fig 3 shows the beam tube measuring point layout and roadway layout.

The beam tube monitoring system laid in the figure is located in air intake lanes 1 and 2. The distance between the measuring points is 20 m, and there are three on each side. The contents of various gases in the goaf during fully mechanized caving face mining were determined using a gas chromatograph provided by the mine.

**Fig 4:**
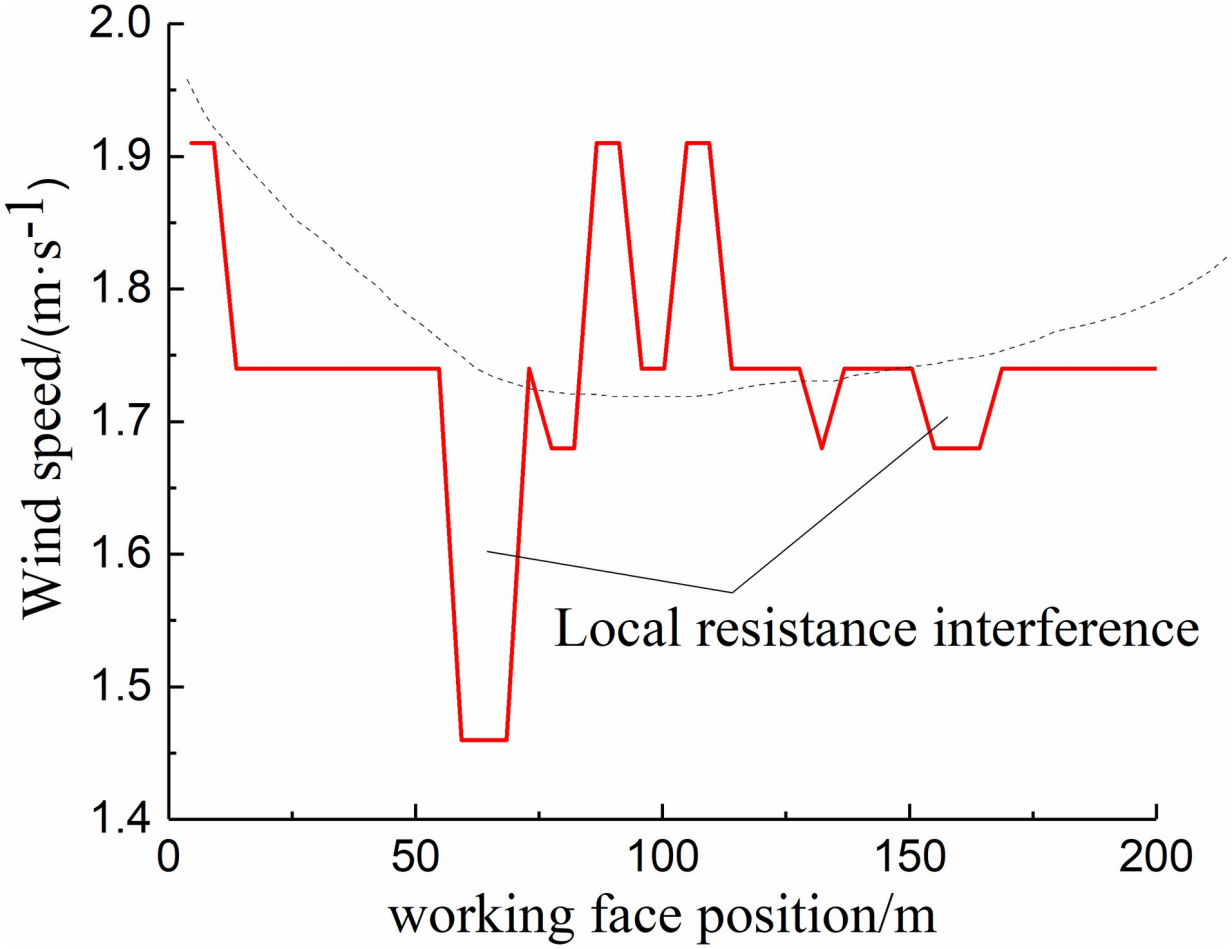
Observation and measurement results of wind speed along the 11101 working face.

**Fig 5:**
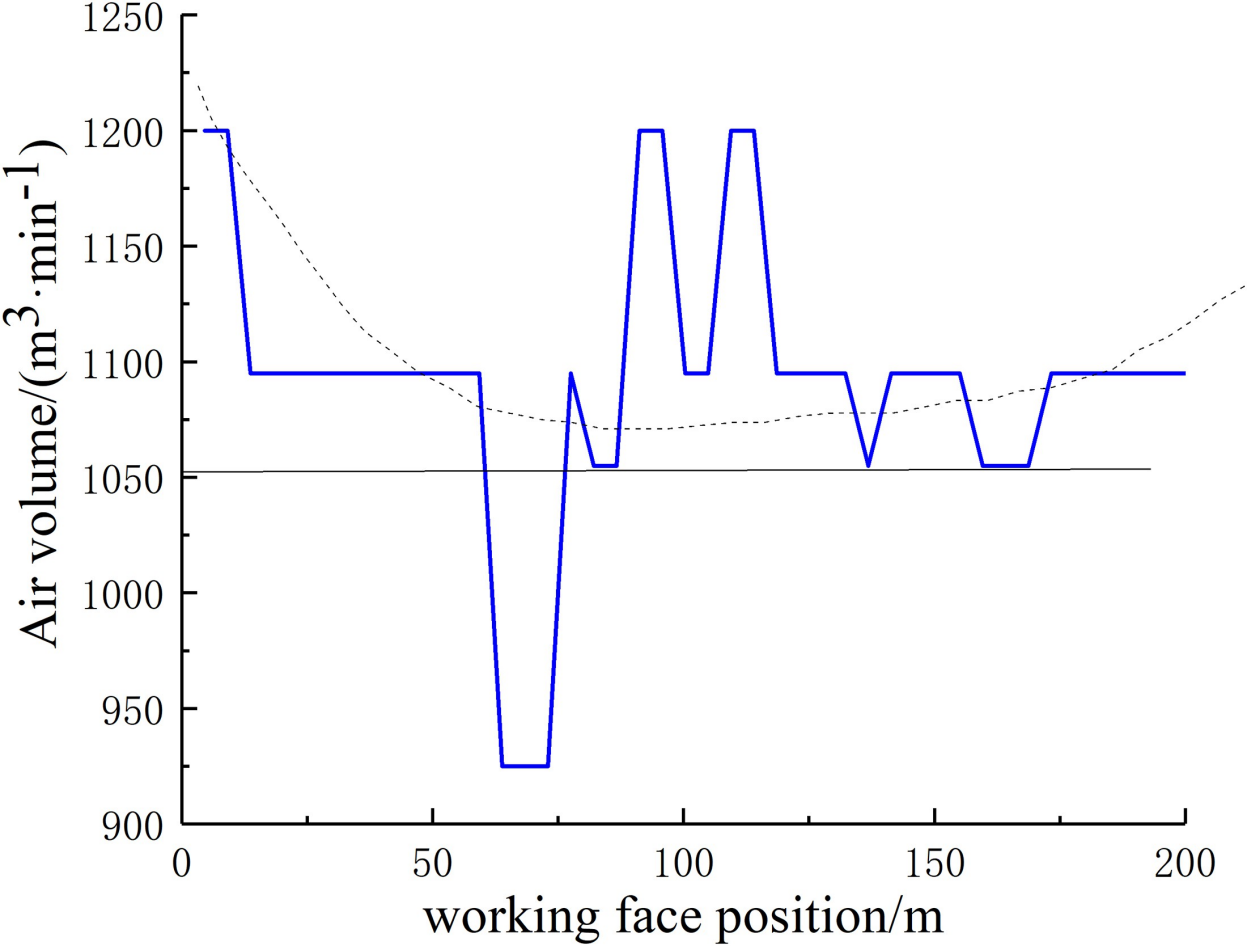
Observation and measurement results of air volume along the 11101 working face.

Based on the data obtained from Qipanjing Mine, the air volume required for the design of the 11101 working face is 1200 m^3^/min. The wind speed curve along the working face of the checkerboard well 11101 was drawn based on the actual measurement results on site, and the air volume curve along the working face of 11101 was drawn accordingly, as shown in Figs 4 and 5. Through the determination of the wind speed, air density, temperature, and other parameters of the working face combined with the determination results of the tilt differential pressure meter, the pressure difference at both ends of the working face was found to be approximately 58.9 Pa.

Based on the results of the closed oxygen consumption experiment, the monitoring results of the 1st side beam pipe in the air intake lane showed that the critical oxygen volume fraction of the suffocating zone inside the goaf behind the working face is approximately 200 m. However, the oxygen volume fraction at each measuring point of the beam tube in the goaf on the second side of the air intake lane is approximately 18%, indicating that the distribution of the oxygen volume fraction continues to extend to the depth of the goaf. In this case, it is impossible to judge the spontaneous combustion risk of the working face.

## IV. Simulation model and boundary conditions

**Fig 6:**
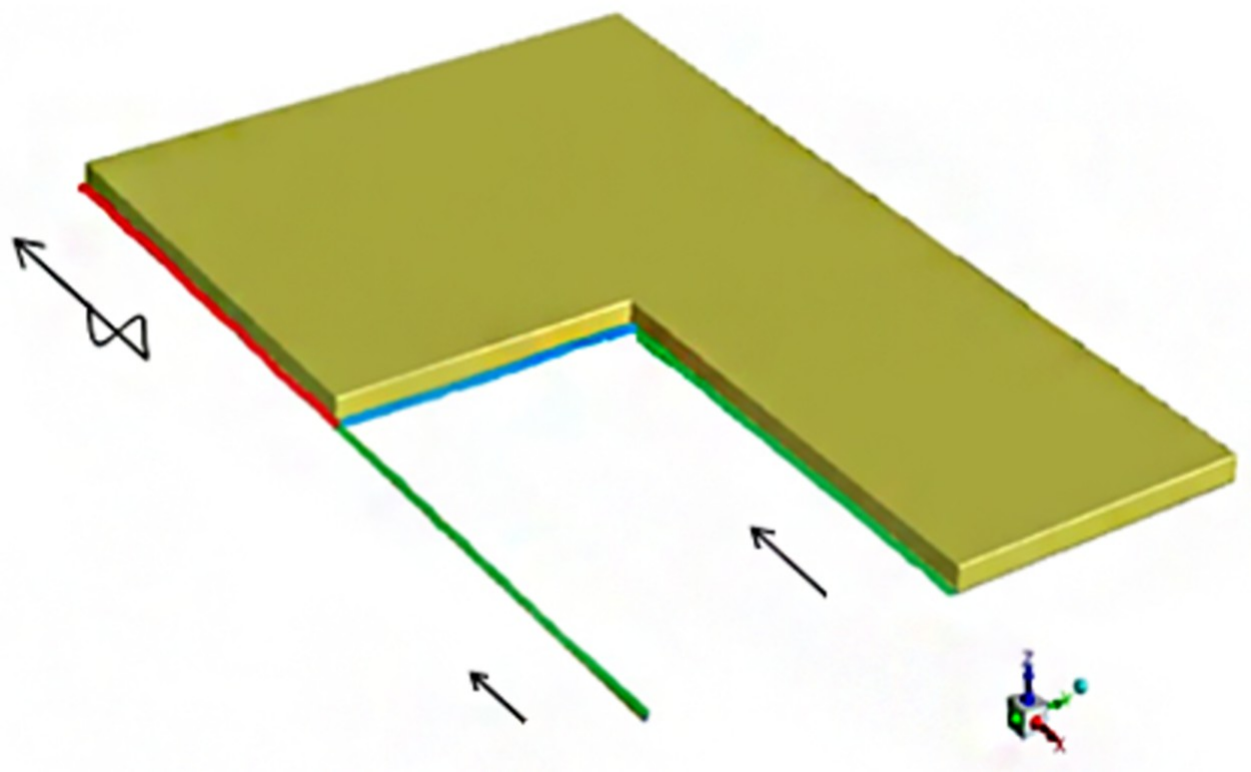
Model drawing of gob-side entry retaining goaf retention in Qipanjing Mine.

Based on on-site measured data, the strike length of the 11101 working face is 2000 m, the dip length is 200 m, the height and width are 3 and 5 m, respectively, and the inlet and return air lanes are 3 m high and 4 m wide. Fig 6 shows one of the established models. According to the “O” ring theory, the caving of the upper goaf and the goaf behind the current working face conforms to a semi-O ring, which is called the “∩+∪” shape: The upper goaf conforms to the shape of “∩”, and the goaf behind the current working face conforms to the shape of “∪.” According to the “O” ring caving theory, a specific control program (UDF) for the caving porous media in the goaf was rewritten [21, 22], and a specific control program (UDF) for the coal waste oxygen consumption was written based on the closed oxygen consumption experiment results. Several groups of model gas emission sources were set.

The simulation of the flow field in the gob-side entry retaining goaf of Qipanjing Mine considers both the air leakage in the rear goaf facing the 11101 working face and the air leakage in the goaf in the upward section of the goaf on the side of the goaf retaining lane (air inlet lane 1). After measuring the air volume of the working face at 20 m^3^/s, the air leakage rate of the working face was determined to be 11% based on the measurement results shown in Figs 4 and 5. To study the influence of the air leakage volume of air intake lane 1 on the goaf along goaf, the air leakage volumes of the lane were set to 0%, 1%, 5%, and 10%, respectively. The oxygen volume fraction of the inlet and return air sides of the goaf behind the current working face was monitored by the beam tube.

## V. Analysis of simulation result

**Fig 7:**
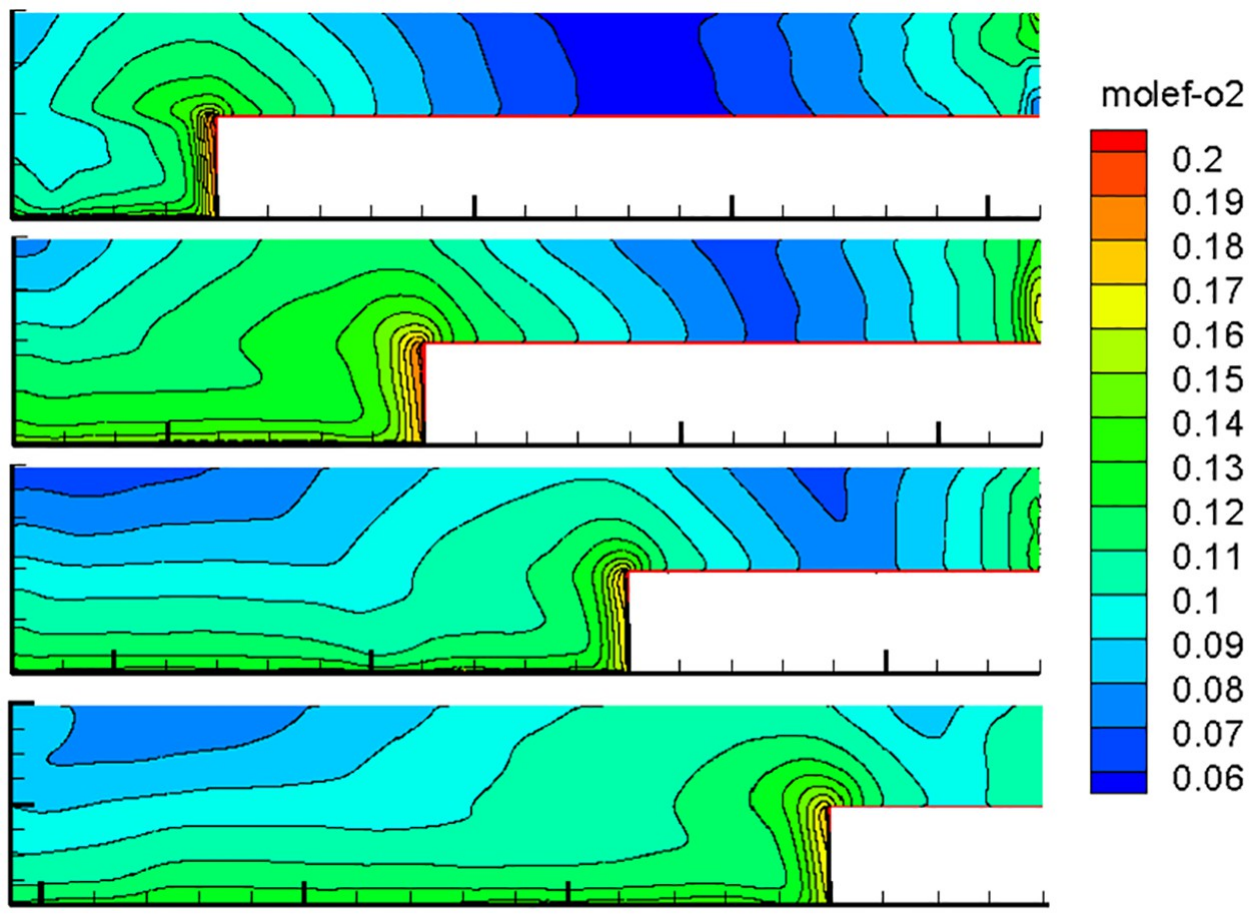
Flow field distribution of the oxygen volume fraction in the goaf at 0% air leakage.

**Fig 8:**
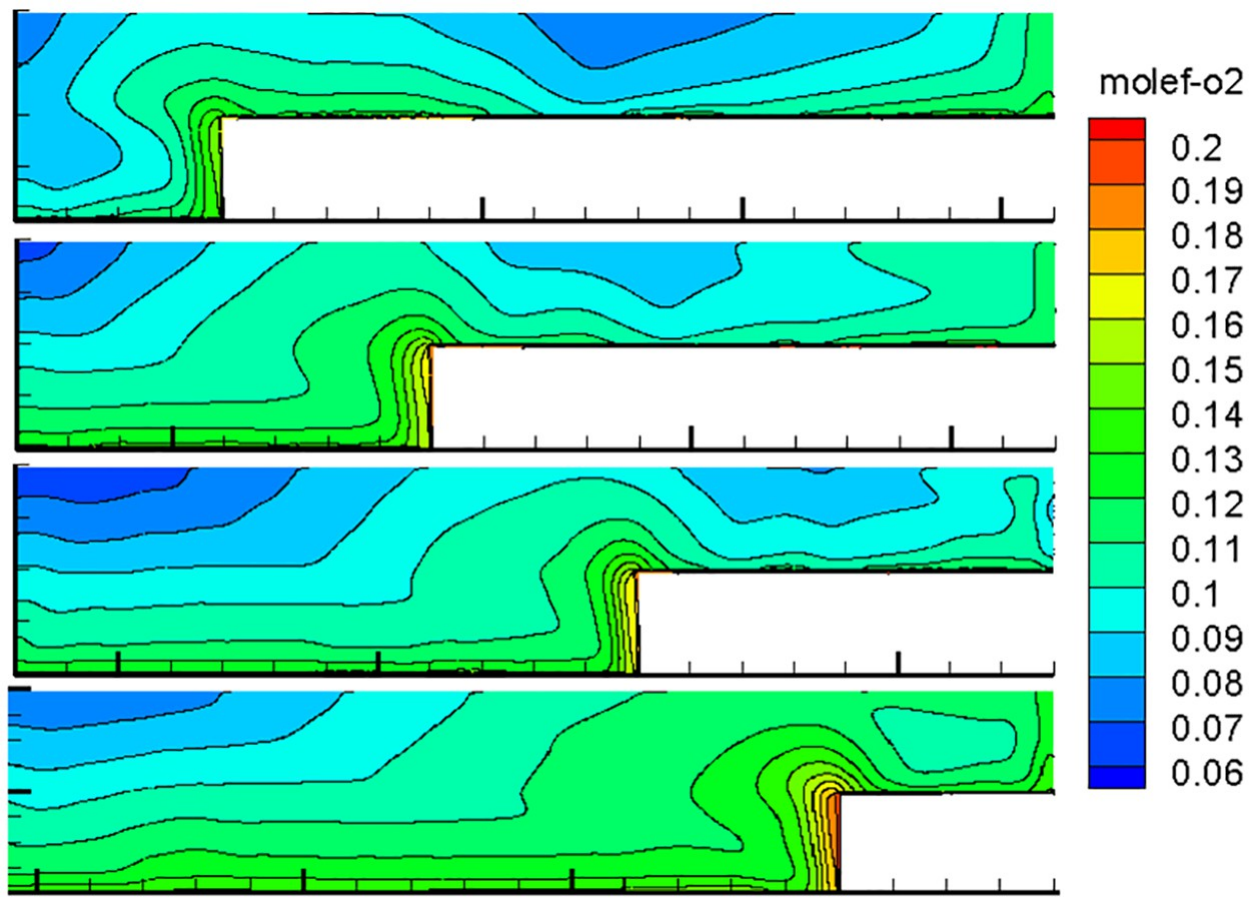
Flow field distribution of the oxygen volume fraction in the goaf at 1% air leakage.

**Fig 9:**
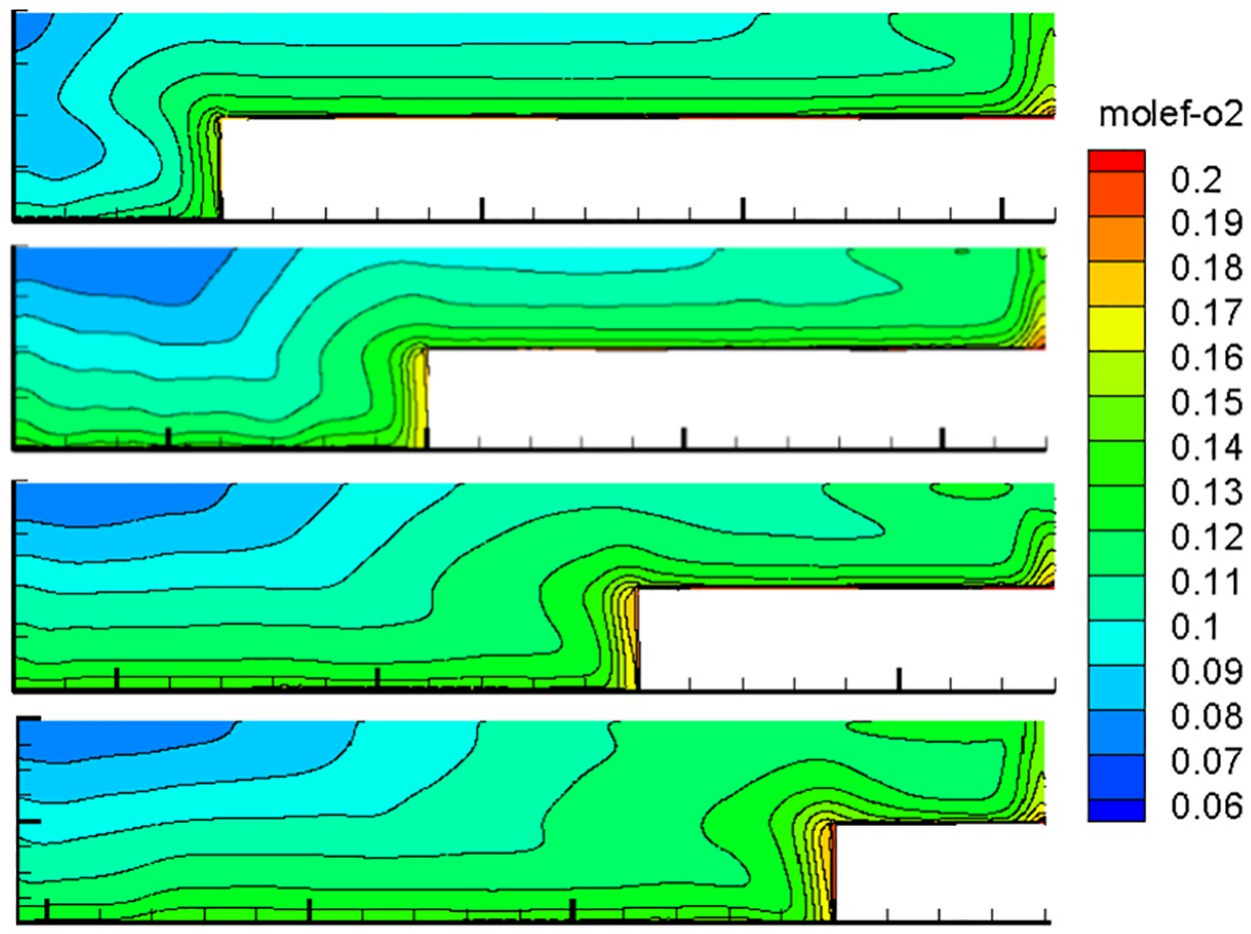
Flow field distribution of the oxygen volume fraction in the goaf at 5% air leakage.

**Fig 10:**
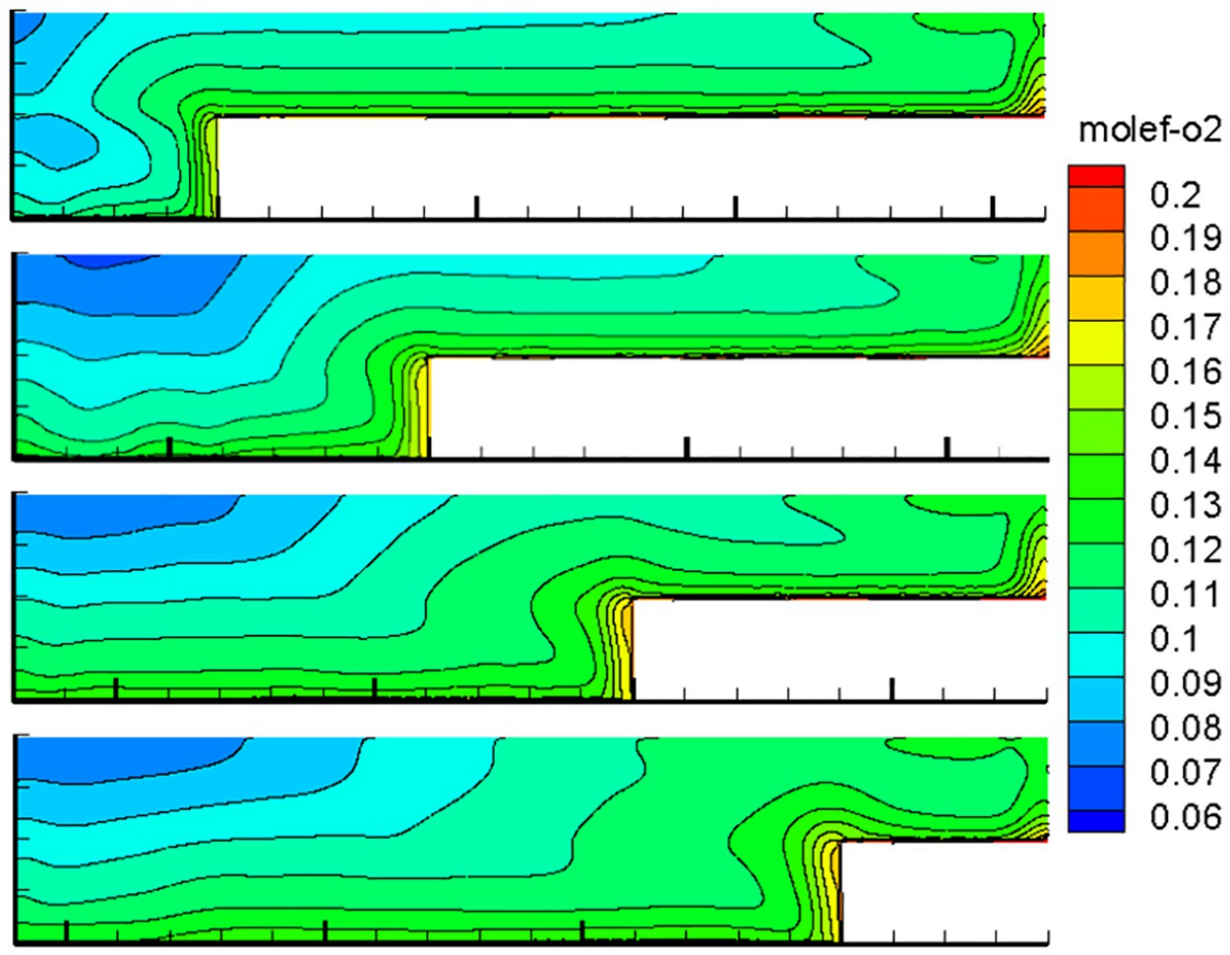
Flow field distribution of the oxygen volume fraction in the goaf at 10% air leakage.

With the advancement of the working face, the length of the inlet and return air roadway changes at all times, and the air leakage of the inlet and return air lanes to the goaf changes constantly, thereby changing the flow field distribution in the goaf with the goaf entry. Therefore, the oxygen flow field distribution in the gob-side entry retaining goaf positions of 400, 600, 800, 1000, 1200, 1400, and 1600 m was simulated. Due to space limitations, only the cloud images of the simulation results with propulsion positions of 400, 800, 1200 and 1600 m are presented herein, as shown in Figs 7–10.

### 1. Flow field distribution in goaf under different conditions

The figures show that the distribution form and range of the oxygen flow field in the goaf vary dynamically with the advancing position of the working face and the air leakage volume in air intake lane 1. When the air leakage volume is the same, the distribution range of the dangerous area in the goaf widens with the advancing length of the working face. With the advancement of the working face, the scope of the goaf and the length of the return air lane increase, while the length of the inlet air lane decreases. The influence of air leakage on the deep mined-out area behind the current working face gradually decreases, whereas its influence on the upper mined-out area gradually increases. Therefore, the distribution of the spontaneous combustion risk areas in the goaf behind the current working face tends to be close to the return air lane, and its proportion in the total area of the goaf decreases. When the air leakage is low, the area of spontaneous combustion risk in the goaf of the upper section decreases, and its proportion increases. However, when the air leakage is high, the oxygen volume fraction in the upper goaf and that in air intake lane 1 of the same length are both greater than 9%, and there is a possibility of spontaneous combustion.

When there is air leakage in the goaf retaining roadway, the oxygen volume fraction in the goaf area near the return air roadway and air inlet roadway 1 is always high (more than 9%), and the residual coal in this area is always in the oxidation state and has a risk of undergoing spontaneous combustion. With the increase in the length of the goaf, the length of this part keeps extending. Therefore, it is impossible to judge the spontaneous combustion risk of the gob-side entry retaining goaf using previous methods.

### 2. Quantitative analysis of simulation results

**Fig 11:**
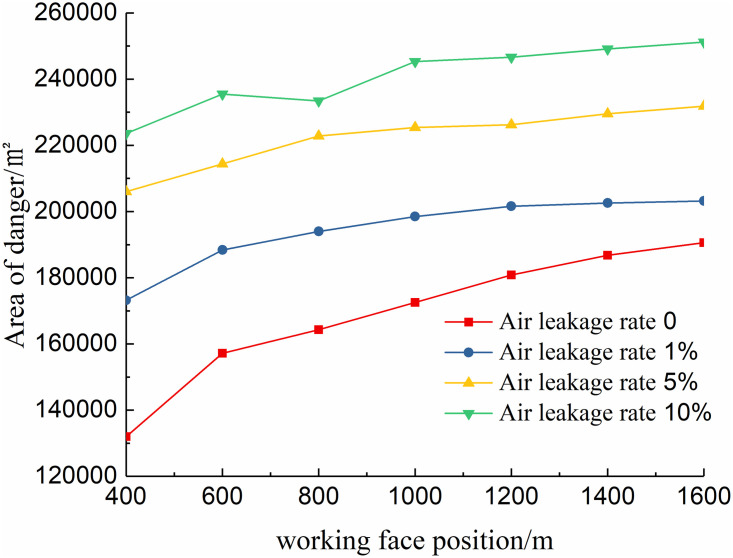
Change curve of the spontaneous combustion danger area under different positions of the working face.

**Fig 12:**
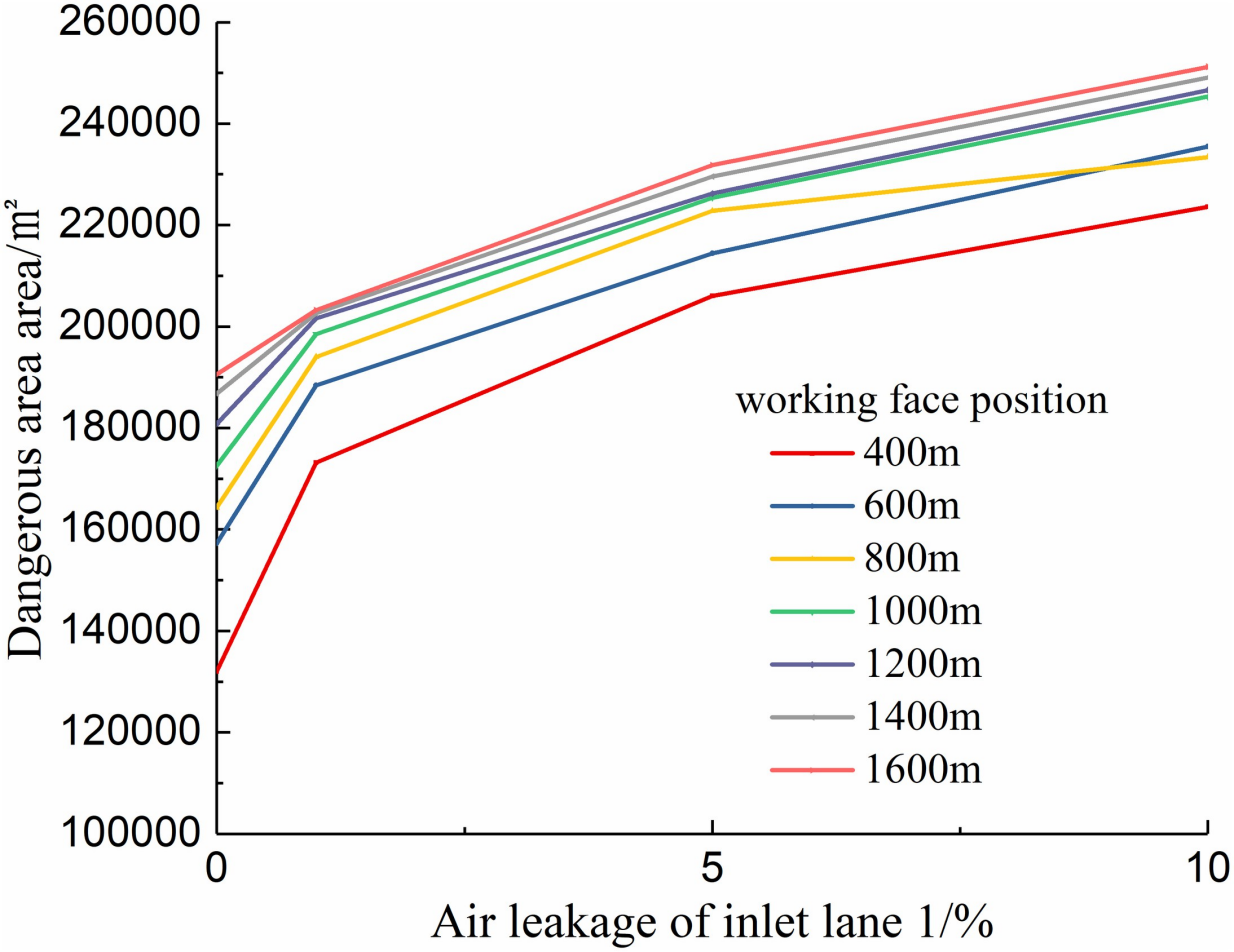
Change curve of the spontaneous combustion danger area under different air leakage.

Quantitatively, in the calculation results, 12 isosurfaces with an oxygen volume fraction greater than 9% and less than 20% were established, and the isosurfaces were then projected onto the Z-axis to obtain the projected area values under different conditions, which are called spontaneous combustion risk areas. In the simulation results, the larger the hazardous area, the higher the possibility of spontaneous combustion in the goaf. Based on the obtained data, the relationship curves of the hazardous area with the advancing position of the working face and the air leakage volume of the entry side (air intake lane 1) were drawn, as shown in Figs 11 and 12.

By fitting the curves shown in Figs 11 and 12, it is found that the curve distribution can be approximated to the power function *S*_*F*_ = *x*^*n*^ *+b* (0 < *n* < 1), where *S*_*F*_ represents the goaf dangerous area, and *x* is the advancing position of the working face or the amount of air leakage.

## VI. Fire prevention measures

The internal situation of the goaf is complicated, making it difficult to predict the specific location and status of fire accurately. In engineering, full-coverage prevention and control measures are often adopted to extinguish the fire due to the combustion of leftover coal in the goaf.

### 1. Fire prevention measures via grouting of return air lane

**Fig 13:**
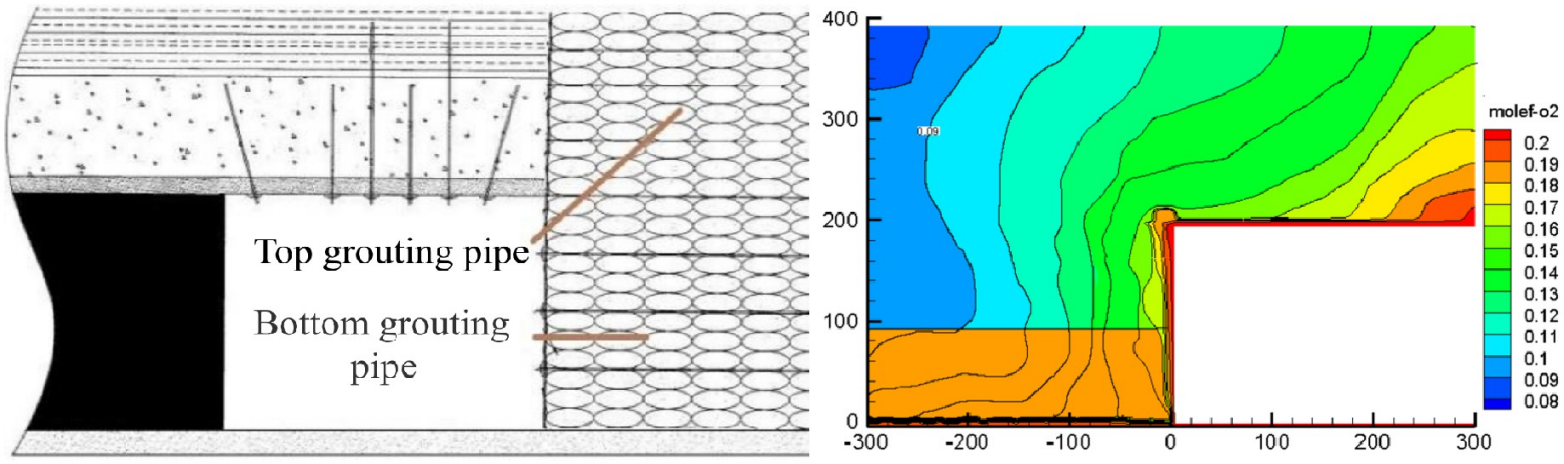
Grouting measures and oxygen distribution in the goaf after ideal grouting.

The leftover coal in a certain range of the goaf is wrapped by grouting to isolate it from oxygen and reduce its oxidation reaction. At the same time, the injected slurry can fill the gap between the floating coal and falling rock to increase its tightness and reduce air leakage. Fig 13 shows the layout and location of the grouting pipe and oxygen distribution in the goaf after ideal grouting.

Under ideal conditions, the width of the natural oxidation zone in the gob-side entry retaining goaf can be determined using the conventional method, and the risk of spontaneous combustion can be judged. The air leakage in the upper section of the goaf can be further reduced when the return air lane in the current section is transformed into the air inlet lane in the lower section.

Based on the method of judging the spontaneous combustion risk of the U-shaped ventilation working face, the spontaneous combustion risk of the goaf in the 11101 working face under 10% air leakage was judged:

$$\tau_{1}^{}=\frac{L_{m}}{\nu_{\text{1}}}.$$ (5)

*L*_*m*_ = 240–20 = 220 m; the results showed that the oxidation time *τ*_*1*_ = 55 d < 66 d for the remaining coal in goaf if the current working face speed is 4 m/d.

When no grouting measure is taken, the oxidation zone and the return air lane are almost parallel in distribution, and the width of the boundary from the working face cannot be determined, making it impossible to determine the spontaneous combustion risk degree of the gob-side entry retaining goaf.

### 2. Measures for side nitrogen injection in air intake lane 1

**Fig 14:**
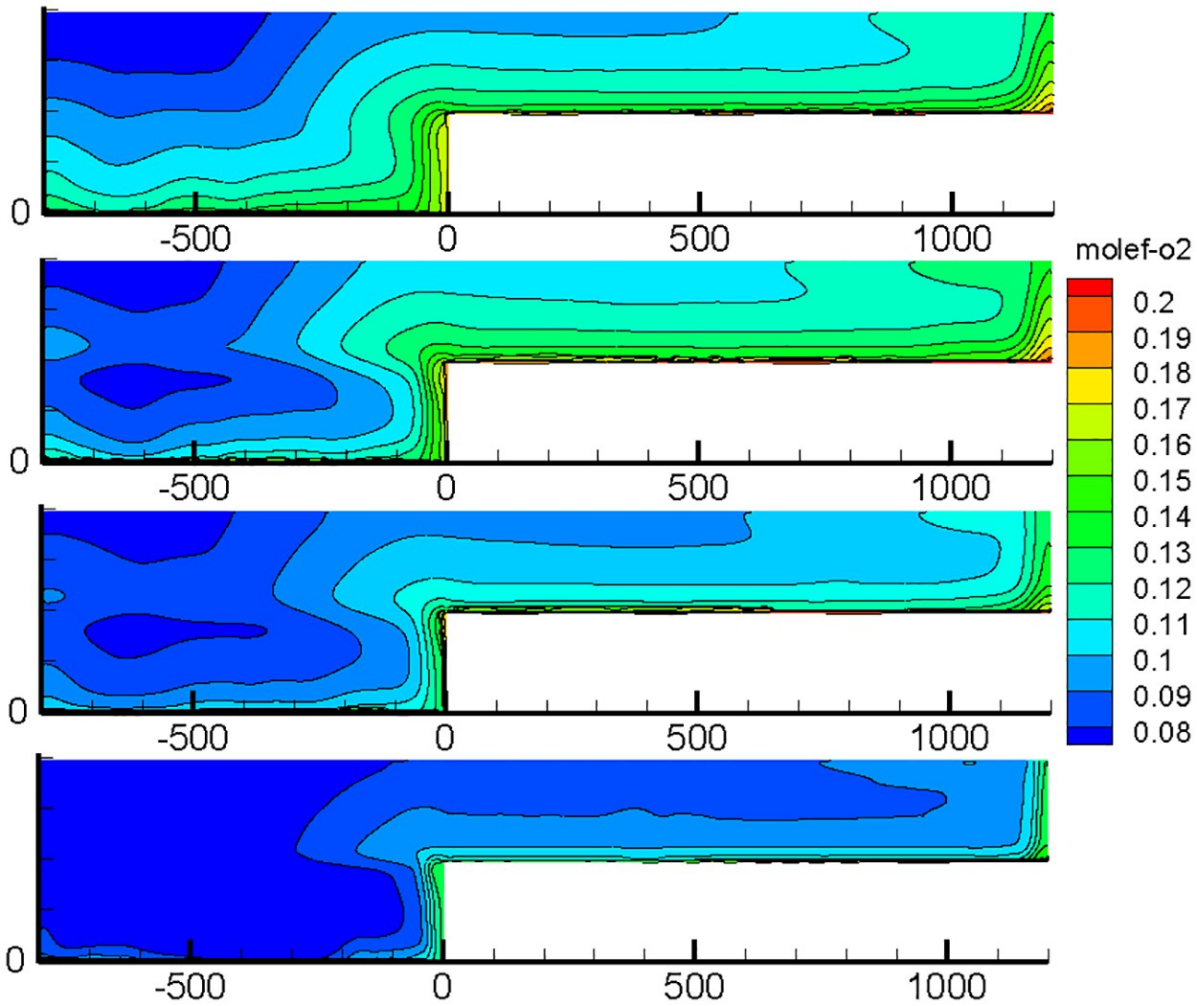
Distribution results of the oxygen flow field in the goaf with different nitrogen injection rates.

In view of the situation of the two air inlet lanes studied, the nitrogen injection location is selected at the position of air inlet lane 1 [23], so as to inert the entire goaf in the form of nitrogen injection through the air leakage flow. To obtain the relationship between the area of the hazardous area and the amount of nitrogen injection, according to <MT/T701-1997 Technical Specification for Nitrogen Fire Prevention and Control in Coal Mines >, the relevant formula for the oxygen content in the oxidation zone of the goaf was calculated, and the amounts of nitrogen required when the amounts of nitrogen injection were 0, 650, 1150, and 1650 m^3^/h; Fig 14 shows the resulting distribution.

**Fig 15:**
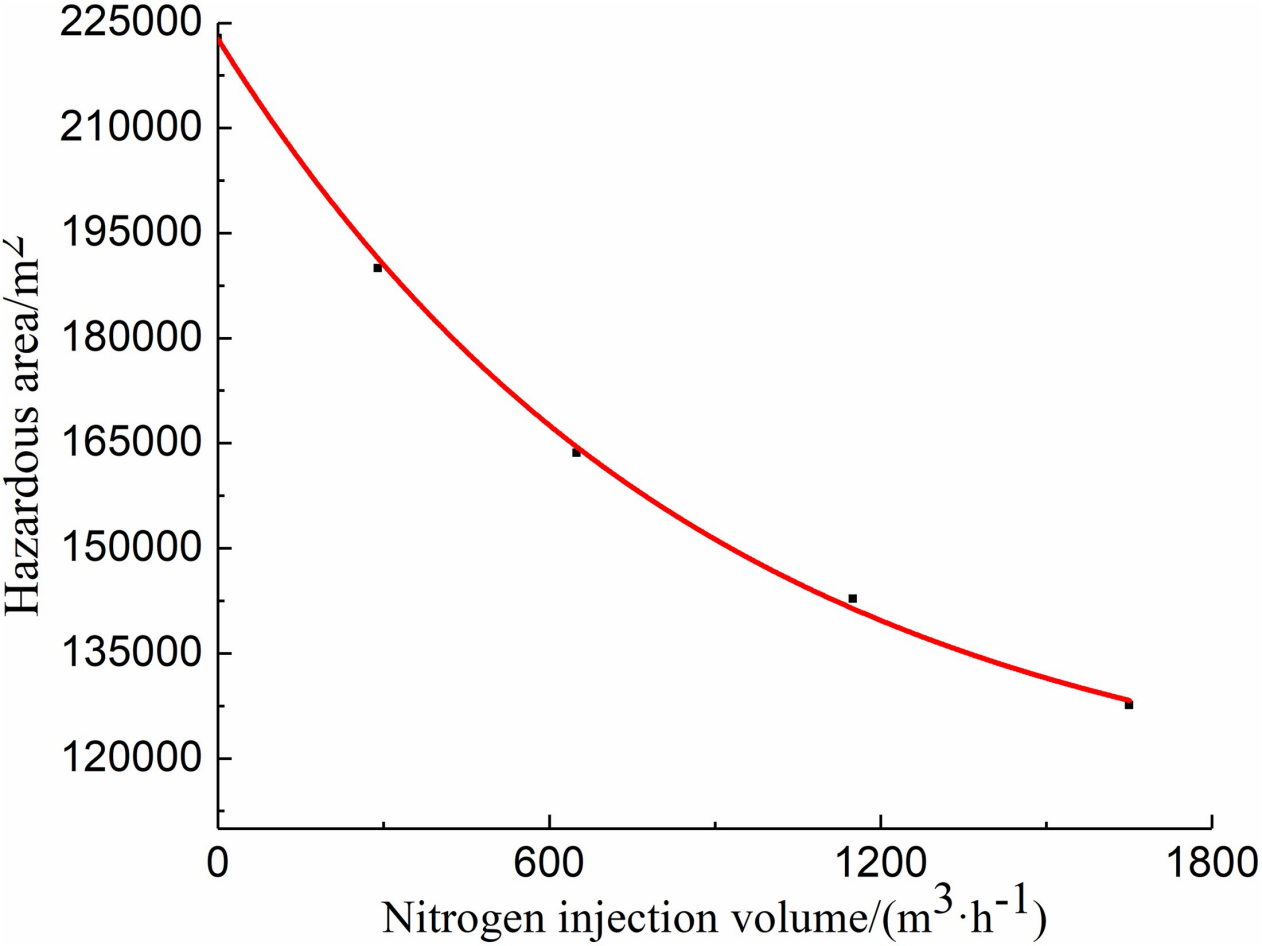
Nitrogen injection effect curve under different nitrogen injection amounts.

The figure shows that the distribution range of the spontaneous combustion risk areas in the goaf decreases with the increase in the nitrogen injection at the entrance of air intake lane 1, and the oxygen volume fraction in the goaf near air intake lane 1 and return lane decreases. For a quantitative analysis, the relationship curve between the hazardous area and the nitrogen injection is also drawn, as shown in Fig 15.

From the curve shown in the figure, it can be inferred that with the increase in the nitrogen injection flow, the hazardous area decreases; however, the decreasing range reduces. This curve can be used to determine the reasonable amount of nitrogen required for injection when the spontaneous combustion risk area is defined or given. This shows that injecting a high nitrogen amount is not conducive; in fact, on the one hand, it will increase the cost, and on the other hand, is less synergistic. An excessive amount of nitrogen injection can easily cause asphyxiation of underground personnel.

## VII. Conclusions

1. Different from coal pillar retaining mining, the distribution of the flow field in the gob-side entry retaining goaf is not relatively constant but constantly changing with the advancement of the working face. The boundary of the spontaneous combustion oxidation zone in the goaf near the return air roadway extends to the depth of the goaf; therefore, the risk of spontaneous combustion in the goaf cannot be judged using the conventional method.
2. The critical oxygen volume fraction and continuous oxygen consumption rate obtained from a closed oxygen consumption experiment are of great significance to judge the danger of spontaneous combustion in the goaf more accurately. With the advancement of the working face or the increase in the air leakage, the spontaneous combustion risk area in the gob-side entry retaining goaf of the Qipanjing Mine was found to increase, but was nonlinear. The fitting analysis showed that the curve distribution can be approximated to a *S*_*F*_ = *x*^*n*^ *+ b* (0 < *n* < 1) function.
3. This study develops on existing methods of fire prevention and extinguishing in the goaf. Two methods were proposed through theoretical verification to help reduce the hazardous area of spontaneous combustion in the gob-side entry retaining goaf. A grouting measure was found to play a role in plugging the leakage and judging the risk of spontaneous combustion in the goaf. The nitrogen injection effect curve obtained can be used as a reference for selecting the nitrogen injection quantity.

## Supporting information

S1 Data (ZIP)
